# Supplementary material for: The experiences of people living with obesity and chronic pain: A Qualitative Evidence Synthesis (QES) protocol
Source: PLoS One. 2024 May 24;19(5):e0302051. doi: 10.1371/journal.pone.0302051 (PMC11125549; doi:10.1371/journal.pone.0302051)
Supplement: S1 Appendix — (PDF) [file pone.0302051.s001.pdf]

The Experiences of People Living with Obesity and Chronic Pain: A Qualitative Evidence Synthesis (QES) Protocol – Hinwood et al. 2024

S1 Appendix 1: Search Strategy

Key Concepts	Population		Study Type
	Chronic Pain	People living with Obesity	Qualitative Evidence
Free text terms	pain	obesity	qualitative
	fibromyalgia	bariatric	QES
	CMP	overweight	interview
	discomfort	BMI	biography
		adiposity	ethnography
		obese	phenomenological
		overeat	perspective
		hyperphagia	narrative*
			hermeneutics
	“musculoskeletal pain”	“weight Loss"	“ethnographic study”
	“chronic pain”	“body mass index”	“focus group*”
		“over weight”	“content analysis”
		“over eat”	“grounded theory”
		“food addiction”	“grounded approach”
			“evidence synthesis”
			“lived experience*”
			“patient-centered care”
			“patient-centred care”
			"patient narrative*"
			"patient stor*"
			“patient experience*”
			“narrative medicine”
			“thematic analysis”
Controlled Vocabulary e.g. MeSH terms	"Pain"[Mesh]	"Body Weight"[Mesh]	"Qualitative Research"[Mesh]
	"Fibromyalgia"[Mesh]	"Overweight"[Mesh]	"Interviews as Topic"[Mesh]
	"Chronic Pain"[Mesh]	"Obesity"[Mesh]	"Focus Groups"[Mesh]
	"Musculoskeletal Pain"[Mesh]	"Bariatrics"[Mesh]	"Grounded Theory"[Mesh]
		"Body Mass Index"[Mesh]	"Narration"[Mesh]
		“Obesity Management"[Mesh]	"Personal Narratives as Topic"[Mesh]
		“Weight Loss"[Mesh]	"Narrative Medicine"[Mesh]
		"Food Addiction"[Mesh]	"Autobiographies as Topic"[Mesh]
			"Patient-Centered Care"[Mesh]

1. PubMed - https://pubmed.ncbi.nlm.nih.gov/advanced/

Search #1	((((((((pain) OR (fibromyalgia)) OR (CMP)) OR (discomfort)) OR ("musculoskeletal pain")) OR ("chronic pain")) OR "Pain"[Mesh]) OR ("Fibromyalgia"[Mesh])) OR ("Chronic Pain"[Mesh])) OR ("Musculoskeletal Pain"[Mesh])
Search #2	((((((((((((((((((obesity) OR (bariatric)) OR (overweight)) OR (BMI)) OR (adiposity)) OR (obese)) OR (overeat)) OR (hyperphagia)) OR ("weight Loss")) OR ("body mass index")) OR ("over weight")) OR ("over eat")) OR ("food addiction")) OR ("Body Weight"[Mesh])) OR ("Overweight"[Mesh])) OR ("Obesity"[Mesh])) OR ("Bariatrics"[Mesh])) OR ("Body Mass Index"[Mesh])) OR ("Obesity Management"[Mesh])) OR ("Weight Loss"[Mesh])) OR ("Food Addiction"[Mesh])
Search #3	((((((((((((((((((((((((((((qualitative) OR (QES)) OR (interview)) OR (biography)) OR (ethnography)) OR (phenomenological)) OR (perspective)) OR (narrative*)) OR (hermeneutics)) OR ("ethnographic study")) OR ("focus group*")) OR ("content analysis")) OR ("grounded theory")) OR ("grounded approach")) OR ("evidence synthesis")) OR ("lived experience*")) OR ("patient-centered care")) OR ("patient-centred care")) OR ("patient narrative*")) OR ("patient stor*")) OR ("patient experience*")) OR ("narrative medicine")) OR ("thematic analysis")) OR ("Qualitative Research"[Mesh])) OR ("Interviews as Topic"[Mesh])) OR ("Focus Groups"[Mesh])) OR ("Grounded Theory"[Mesh])) OR ("Narration"[Mesh])) OR ("Personal Narratives as Topic"[Mesh])) OR ("Narrative Medicine"[Mesh])) OR ("Autobiographies as Topic"[Mesh])) OR ("Patient-Centered Care"[Mesh])
Search #4	Search #1 AND Search #2 AND Search #3
	((((((((((pain) OR (fibromyalgia)) OR (CMP)) OR (discomfort)) OR ("musculoskeletal pain")) OR ("chronic pain")) OR "Pain"[Mesh]) OR ("Fibromyalgia"[Mesh])) OR ("Chronic Pain"[Mesh])) OR ("Musculoskeletal Pain"[Mesh])) AND ((((((((((((((((((((obesity) OR (bariatric)) OR (overweight)) OR (BMI)) OR (adiposity)) OR (obese)) OR (overeat)) OR (hyperphagia)) OR ("weight Loss")) OR ("body mass index")) OR ("over weight")) OR ("over eat")) OR ("food addiction")) OR ("Body Weight"[Mesh])) OR ("Overweight"[Mesh])) OR ("Obesity"[Mesh])) OR ("Bariatrics"[Mesh])) OR ("Body Mass Index"[Mesh])) OR ("Obesity Management"[Mesh])) OR ("Weight Loss"[Mesh])) OR ("Food Addiction"[Mesh]))) AND ((((((((((((((((((((((((((((((qualitative) OR (QES)) OR (interview)) OR (biography)) OR (ethnography)) OR (phenomenological)) OR (perspective)) OR (narrative*)) OR (hermeneutics)) OR ("ethnographic study")) OR ("focus group*")) OR ("content analysis")) OR ("grounded theory")) OR ("grounded approach")) OR ("evidence synthesis")) OR ("lived experience*")) OR ("patient-centered care")) OR ("patient-centred care")) OR ("patient narrative*")) OR ("patient stor*")) OR ("patient experience*")) OR ("narrative medicine")) OR ("thematic analysis")) OR ("Qualitative Research"[Mesh])) OR ("Interviews as Topic"[Mesh])) OR ("Focus Groups"[Mesh])) OR ("Grounded Theory"[Mesh])) OR ("Narration"[Mesh])) OR ("Personal Narratives as Topic"[Mesh])) OR ("Narrative Medicine"[Mesh])) OR ("Autobiographies as Topic"[Mesh])) OR ("Patient-Centered Care"[Mesh]))

2. Embase - https://www.embase.com/#advancedSearch/resultspage/history.7/page.1/25.items/orderby.date/source.

Search #1	'pain'/exp OR pain OR 'fibromyalgia'/exp OR fibromyalgia OR 'cmp'/exp OR cmp OR 'discomfort'/exp OR discomfort OR 'musculoskeletal pain'/exp OR 'musculoskeletal pain' OR 'chronic pain'/exp OR 'chronic pain'
Search #2	'obesity'/exp OR obesity OR bariatric OR 'overweight'/exp OR overweight OR 'bmi'/exp OR bmi OR 'adiposity'/exp OR adiposity OR obese OR overeat OR 'hyperphagia'/exp OR hyperphagia OR 'weight loss'/exp OR 'weight loss' OR 'body mass index'/exp OR 'body mass index' OR 'over weight' OR 'over eat' OR 'food addiction'/exp OR 'food addiction'

Search #3	'qualitative'/exp OR qualitative OR qes OR 'interview'/exp OR interview OR 'biography'/exp OR biography OR 'ethnography'/exp OR ethnography OR phenomenological OR 'perspective'/exp OR perspective OR narrative* OR 'hermeneutics'/exp OR hermeneutics OR 'ethnographic study' OR 'focus group*' OR 'content analysis'/exp OR 'content analysis' OR 'grounded theory'/exp OR 'grounded theory' OR 'grounded approach' OR 'evidence synthesis'/exp OR 'evidence synthesis' OR 'lived experience*' OR 'patient-centered care'/exp OR 'patient-centered care' OR 'patient-centred care' OR 'patient narrative*' OR 'patient stor*' OR 'patient experience*' OR 'narrative medicine'/exp OR 'narrative medicine' OR 'thematic analysis'
Search #4	Search #1 AND Search #2 AND Search #3
	('pain'/exp OR pain OR 'fibromyalgia'/exp OR fibromyalgia OR 'cmp'/exp OR cmp OR 'discomfort'/exp OR discomfort OR 'musculoskeletal pain'/exp OR 'musculoskeletal pain' OR 'chronic pain'/exp OR 'chronic pain') AND ('obesity'/exp OR obesity OR bariatric OR 'overweight'/exp OR overweight OR 'bmi'/exp OR bmi OR 'adiposity'/exp OR adiposity OR obese OR overeat OR 'hyperphagia'/exp OR hyperphagia OR 'weight loss'/exp OR 'weight loss' OR 'body mass index'/exp OR 'body mass index' OR 'over weight' OR 'over eat' OR 'food addiction'/exp OR 'food addiction') AND ('qualitative'/exp OR qualitative OR qes OR 'interview'/exp OR interview OR 'biography'/exp OR biography OR 'ethnography'/exp OR ethnography OR phenomenological OR 'perspective'/exp OR perspective OR narrative* OR 'hermeneutics'/exp OR hermeneutics OR 'ethnographic study' OR 'focus group*' OR 'content analysis'/exp OR 'content analysis' OR 'grounded theory'/exp OR 'grounded theory' OR 'grounded approach' OR 'evidence synthesis'/exp OR 'evidence synthesis' OR 'lived experience*' OR 'patient-centered care'/exp OR 'patient-centered care' OR 'patient-centred care' OR 'patient narrative*' OR 'patient stor*' OR 'patient experience*' OR 'narrative medicine'/exp OR 'narrative medicine' OR 'thematic analysis')

3. Web of Science - https://www-webofscience-com.ucd.idm.oclc.org/wos/woscc/advanced-search

Search #1	((((ALL=(pain)) OR ALL=(fibromyalgia)) OR ALL=(CMP)) OR ALL=(discomfort)) OR ALL=("musculoskeletal pain")) OR ALL=("chronic pain")
Search #2	((((((((((ALL=(obesity)) OR ALL=(bariatric)) OR ALL=(overweight)) OR ALL=(BMI)) OR ALL=(adiposity)) OR ALL=(obese)) OR ALL=(overeat)) OR ALL=(hyperphagia)) OR ALL=("weight Loss")) OR ALL=("body mass index")) OR ALL=("over weight")) OR ALL=("over eat")) OR ALL=("food addiction")
Search #3	((((((((((((((((((ALL=(qualitative)) OR ALL=(QES)) OR ALL=(interview)) OR ALL=(biography)) OR ALL=(ethnography)) OR ALL=(phenomenological)) OR ALL=(perspective)) OR ALL=(narrative*)) OR ALL=(hermeneutics)) OR ALL=("ethnographic study")) OR ALL=("focus group*")) OR ALL=("content analysis")) OR ALL=("grounded theory")) OR ALL=("grounded approach")) OR ALL=("evidence synthesis")) OR ALL=("lived experience*")) OR ALL=("patient-centered care")) OR ALL=("patient-centred care")) OR ALL=("patient narrative*")) OR ALL=("patient stor*")) OR ALL=("patient experience*")) OR ALL=("narrative medicine")
Search #4	Search #1 AND Search #2 AND Search #3 https://www.webofscience.com/wos/woscc/summary/cbf475b0-35f8-497d-884c-b2bfde8ad74e-6f5df39b/relevance/1

	#3 AND #2 AND #1

4. CINAHL - https://web-s-ebscohost-com.ucd.idm.oclc.org/ehost/search/advanced?vid=15&sid=5aa872f3-16ab-4608-954b-e1264d170aec%40redis

Search #1	((((((((pain) OR (fibromyalgia)) OR (CMP)) OR (discomfort)) OR ("musculoskeletal pain")) OR ("chronic pain")) OR "Pain"[Mesh]) OR ("Fibromyalgia"[Mesh])) OR ("Chronic Pain"[Mesh])) OR ("Musculoskeletal Pain"[Mesh])
Search #2	(((((((((((((((((obesity) OR (bariatric)) OR (overweight)) OR (BMI)) OR (adiposity)) OR (obese)) OR (overeat)) OR (hyperphagia)) OR ("weight Loss")) OR ("body mass index")) OR ("over weight")) OR ("over eat")) OR ("food addiction")) OR ("Body Weight"[Mesh])) OR ("Overweight"[Mesh])) OR ("Obesity"[Mesh])) OR ("Bariatrics"[Mesh])) OR ("Body Mass Index"[Mesh])) OR ("Obesity Management"[Mesh])) OR ("Weight Loss"[Mesh])) OR ("Food Addiction"[Mesh])
Search #3	((((((((((((((((((((((((((((qualitative) OR (QES)) OR (interview)) OR (biography)) OR (ethnography)) OR (phenomenological)) OR (perspective)) OR (narrative*)) OR (hermeneutics)) OR ("ethnographic study")) OR ("focus group*")) OR ("content analysis")) OR ("grounded theory")) OR ("grounded approach")) OR ("evidence synthesis")) OR ("lived experience*")) OR ("patient-centered care")) OR ("patient-centred care")) OR ("patient narrative*")) OR ("thematic analysis")) OR ("patient stor*")) OR ("patient experience*")) OR ("narrative medicine")) OR ("Qualitative Research"[Mesh])) OR ("Interviews as Topic"[Mesh])) OR ("Focus Groups"[Mesh])) OR ("Grounded Theory"[Mesh])) OR ("Narration"[Mesh])) OR ("Personal Narratives as Topic"[Mesh])) OR ("Narrative Medicine"[Mesh])) OR ("Autobiographies as Topic"[Mesh])) OR ("Patient-Centered Care"[Mesh])
Search #4	Search #1 AND Search #2 AND Search #3
	((((((((((pain) OR (fibromyalgia)) OR (CMP)) OR (discomfort)) OR ("musculoskeletal pain")) OR ("chronic pain")) OR "Pain"[Mesh]) OR ("Fibromyalgia"[Mesh])) OR ("Chronic Pain"[Mesh])) OR ("Musculoskeletal Pain"[Mesh])) AND ((((((((((((((((((((obesity) OR (bariatric)) OR (overweight)) OR (BMI)) OR (adiposity)) OR (obese)) OR (overeat)) OR (hyperphagia)) OR ("weight Loss")) OR ("body mass index")) OR ("over weight")) OR ("over eat")) OR ("food addiction")) OR ("Body Weight"[Mesh])) OR ("Overweight"[Mesh])) OR ("Obesity"[Mesh])) OR ("Bariatrics"[Mesh])) OR ("Body Mass Index"[Mesh])) OR ("Obesity Management"[Mesh])) OR ("Weight Loss"[Mesh])) OR ("Food Addiction"[Mesh]))) AND (((((((((((((((((((((((((((((((qualitative) OR (QES)) OR (interview)) OR (biography)) OR (ethnography)) OR (phenomenological)) OR (perspective)) OR (narrative*)) OR (hermeneutics)) OR ("ethnographic study")) OR ("focus group*")) OR ("thematic analysis")) OR ("content analysis")) OR ("grounded theory")) OR ("grounded approach")) OR ("evidence synthesis")) OR ("lived experience*")) OR ("patient-centered care")) OR ("patient-centred care")) OR ("patient narrative*")) OR ("patient stor*")) OR ("patient experience*")) OR ("narrative medicine")) OR ("Qualitative Research"[Mesh])) OR ("Interviews as Topic"[Mesh])) OR ("Focus Groups"[Mesh])) OR ("Grounded Theory"[Mesh])) OR ("Narration"[Mesh])) OR ("Personal Narratives as Topic"[Mesh])) OR ("Narrative Medicine"[Mesh])) OR ("Autobiographies as Topic"[Mesh])) OR ("Patient-Centered Care"[Mesh]))

5. PsycInfo - https://web-p-ebscohost-com.ucd.idm.oclc.org/ehost/search/advanced?vid=0&sid=41630e60-61ef-4082-98ec-09c465c90f90%40redis

Search #1	((((((((pain) OR (fibromyalgia)) OR (CMP)) OR (discomfort)) OR ("musculoskeletal pain")) OR ("chronic pain")) OR "Pain"[Mesh]) OR ("Fibromyalgia"[Mesh])) OR ("Chronic Pain"[Mesh])) OR ("Musculoskeletal Pain"[Mesh])
Search #2	((((((((((((((((((obesity) OR (bariatric)) OR (overweight)) OR (BMI)) OR (adiposity)) OR (obese)) OR (overeat)) OR (hyperphagia)) OR ("weight Loss")) OR ("body mass index")) OR ("over weight")) OR ("over eat")) OR ("food addiction")) OR ("Body Weight"[Mesh])) OR ("Overweight"[Mesh])) OR ("Obesity"[Mesh])) OR ("Bariatrics"[Mesh])) OR ("Body Mass Index"[Mesh])) OR ("Obesity Management"[Mesh])) OR ("Weight Loss"[Mesh])) OR ("Food Addiction"[Mesh])
Search #3	((((((((((((((((((((((((((((((qualitative) OR (QES)) OR (interview)) OR (biography)) OR (ethnography)) OR (phenomenological)) OR (perspective)) OR (narrative*)) OR (hermeneutics)) OR ("ethnographic study")) OR ("focus group*")) OR ("content analysis")) OR ("grounded theory")) OR ("grounded approach")) OR ("evidence synthesis")) OR ("lived experience*")) OR ("patient-centered care")) OR ("patient-centred care")) OR ("patient narrative*")) OR ("thematic analysis")) OR ("patient stor*")) OR ("patient experience*")) OR ("narrative medicine")) OR ("Qualitative Research"[Mesh])) OR ("Interviews as Topic"[Mesh])) OR ("Focus Groups"[Mesh])) OR ("Grounded Theory"[Mesh])) OR ("Narration"[Mesh])) OR ("Personal Narratives as Topic"[Mesh])) OR ("Narrative Medicine"[Mesh])) OR ("Autobiographies as Topic"[Mesh])) OR ("Patient-Centered Care"[Mesh])
Search #4	Search #1 AND Search #2 AND Search #3<br>((((((((((pain) OR (fibromyalgia)) OR (CMP)) OR (discomfort)) OR ("musculoskeletal pain")) OR ("chronic pain")) OR "Pain"[Mesh]) OR ("Fibromyalgia"[Mesh])) OR ("Chronic Pain"[Mesh])) OR ("Musculoskeletal Pain"[Mesh])) AND (((((((((((((((((((obesity) OR (bariatric)) OR (overweight)) OR (BMI)) OR (adiposity)) OR (obese)) OR (overeat)) OR (hyperphagia)) OR ("weight Loss")) OR ("body mass index")) OR ("over weight")) OR ("over eat")) OR ("food addiction")) OR ("Body Weight"[Mesh])) OR ("Overweight"[Mesh])) OR ("Obesity"[Mesh])) OR ("Bariatrics"[Mesh])) OR ("Body Mass Index"[Mesh])) OR ("Obesity Management"[Mesh])) OR ("Weight Loss"[Mesh])) OR ("Food Addiction"[Mesh]))) AND ((((((((((((((((((((((((((((((((qualitative) OR (QES)) OR (interview)) OR (biography)) OR (ethnography)) OR (phenomenological)) OR (perspective)) OR (narrative*)) OR (hermeneutics)) OR ("ethnographic study")) OR ("focus group*")) OR ("thematic analysis")) OR ("content analysis")) OR ("grounded theory")) OR ("grounded approach")) OR ("evidence synthesis")) OR ("lived experience*")) OR ("patient-centered care")) OR ("patient-centred care")) OR ("patient narrative*")) OR ("patient stor*")) OR ("patient experience*")) OR ("narrative medicine")) OR ("Qualitative Research"[Mesh])) OR ("Interviews as Topic"[Mesh])) OR ("Focus Groups"[Mesh])) OR ("Grounded Theory"[Mesh])) OR ("Narration"[Mesh])) OR ("Personal Narratives as Topic"[Mesh])) OR ("Narrative Medicine"[Mesh])) OR ("Autobiographies as Topic"[Mesh])) OR ("Patient-Centered Care"[Mesh]))
