# Supplementary material for: The experiences of people living with obesity and chronic pain: A Qualitative Evidence Synthesis (QES) protocol
Source: PLoS One. 2024 May 24;19(5):e0302051. doi: 10.1371/journal.pone.0302051 (PMC11125549; doi:10.1371/journal.pone.0302051)
Supplement: Attachment — Submitted filename: Response to reviewers PLOSONE 19.01.24 NH.docx [file pone.0302051.s003.docx]

**Response to Editorial Team and Reviewer Comments**

**Reference**: PONE-D-23-17779

**Title**: The Experiences of People Living with Obesity and Chronic Pain: A Qualitative Evidence Synthesis (QES) Protocol

**Journal**: PLOS ONE

**Corresponding author:** Natasha S. Hinwood

**Address**: UCD School of Public Health, Physiotherapy and Sport Science, University College Dublin, Dublin, Ireland

**Email**: [natasha.hinwood@ucdconnect.ie](mailto:natasha.hinwood@ucdconnect.ie)

**Phone**: +353 1 716 6511

Dear Editorial Team,

We are very grateful to you and the reviewers for all your time and attention to our manuscript. Please find below our responses to the points raised by the two reviewers during the review process.

Kindest regards,

Natasha Hinwood

*Black text italicised = reviewer comments*

Blue text = authors response

Black text = text in protocol

Red text = amendments to protocol text

***Reviewer #1:***

*The paper is simply the description of PRISMA guidelines, and even made incompletely. I may raise several questions to authors:
1) What is the scientific sense to publish such paper?*

*2) In which manner it increase the knowledge of the readership?*

*I think that with out results and complete discussion of such paper it is useless to publish, especially if the the protocol has already been registered and described in the PROSPERO registry.*

We thank you for your comments. As you mention and as we have clarified in the submitted manuscript, this study has been pre-registered with PROSPERO. There are several reasons we feel publication of the manuscript is warranted. These are as follows:

- The submitted protocol manuscript provides a more detailed description of the study's methods, beyond that available in the PROSPERO protocol and the final paper (typically limited by word count restrictions). Publishing richer detail regarding methodology and data synthesis plans enhances reproducibility and independent verification of results, in turn enhancing validity of results.
- Publication of the protocol in a reputable peer-review publication, such as PlosONE, broadens the potential audience and awareness for this research. This increases the accessibility, discoverability, and potential impact of the study. Ultimately, this may help to avoid duplication of similar studies, consolidate efforts and better coordinate research activity.
- Publishing this research protocol in this substantive manner ensures and facilitates transparency of this work for other researchers and members of the public. Fundamentally, improved transparency helps to facilitate further trust in research.
- The publication of protocols helps to reduce publication bias.
- Finally, the publication of the fuller manuscript facilitates early feedback via peer review, both during the publication process (i.e. presently) and post-publication as described previously.

***Reviewer #2:*** *1. Please define abbreviations upon first appearance in the text.*

We thank you for your comment. We have checked all abbreviations and amended the text as follows:

The disease of obesity and disorder of chronic musculoskeletal pain (CMP) are both complex, and weight loss treatments are only a part of the treatment continuum [42,43, 44].

*2. Line 129: please list the five sites in question.*

We thank you for your comment. We have amended the text as follows. Amendments are noted in red text.

Previously:

A Swedish obesity registry study reported a pain prevalence (pain in at least one of five body locations) of 58% among men and 68% among women [19].

Amended to:

A Swedish obesity registry study reported a pain prevalence (pain in five body locations: neck, back, hip, knee and ankles) of 58% among men and 68% among women [19].

*3. Line 131: the formulation of the sentence is ambiguous to the reader.*

We thank you for your comment. We have amended the text as follows. Amendments are noted in red text.

Previously:

People living with obesity and pain have a higher pain incidence, severity and morbidity, with substantially decreased health-related quality of life when compared to those living with either condition alone [11,20].

Amended to:

People living with obesity and pain have a higher pain incidence, severity and morbidity. This population also experiences substantially decreased health-related quality of life when compared to those living with either condition alone [11,20].

*4. Why the authors didn’t include the Cochrane library to the list of databases to be searched?*

To our knowledge, the Cochrane library does not typically contain primary qualitative studies so was not included. We were confident of the validity of our included databases.

*5. Why the authors won’t include unpublished studies? Omitting these studies may create bias.*

We did discuss this, however we decided to only include studies that had undergone peer review as a form of quality assurance. We accept that sources of grey literature could have potentially identified additional eligible studies, nevertheless we similarly chose to exclude studies not published in English. We have added a comment regarding implications of this to the Article Summary section, under Limitations:

“The exclusion of studies not published in English or peer reviewed (such as grey literature) may exclude some data and limit the generalisability of our findings.”

*6. Please specify the patients’ characteristics that will be extracted. Will it include the site of pain; duration of pain; level of obesity etc., …?*

We thank you for your comment. We have amended the text as follows. Amendments are noted in red text.

“A proforma devised for the review will allow for descriptive data to be captured from the included studies : First author, author characteristics, year of publication, country of origin, study funding, conflicts of interest, ethical approval, study aims, study design, rationale for study and theoretical background, study duration, study setting, sampling and recruitment methods, study inclusion and exclusion criteria, final sample size, method(s) of data collection, use of inductive or deductive coding approaches, data analysis method, themes (and subthemes) identified, and participant characteristics (including age, gender, ethnicity, employment status, BMI characteristics and pain characteristics). For the BMI or weight characteristics, we will aim to extract any information relating to the BMI of included participants from each study, including descriptive statistics (range, mean, standard deviation) where possible. For patient pain characteristics, we will extract information about the severity, duration, aetiology, diagnosis, relevant previous treatment, and location of pain, where available, for participants included in each study.”

*7. Will the authors conduct subgroup analysis to assess heterogeneity of findings? This analysis should be described in detail.*

As a qualitative (rather than quantitative) evidence synthesis, we have not planned any *a priori* subgroup analyses. If on full text review and synthesis the qualitative data suggest that any subgroup analyses are merited, we will consider this and report any subgroup analyses as *post hoc* decisions. We have however added the following text to the manuscript.

“If during the analysis, there are any emergent subgroups (e.g. based on participant characteristics, type of pain, BMI category, or setting), these will be considered for further subgroup analysis. Any identified subgroup data will follow the same synthesis plan as outlined above. Any subgroup analyses will be reported as post hoc decisions.”
